# Opium Smoking

**Published:** 1848-02

**Authors:** 


*Opium Smoking.*—Opium is prepared for smoking much in the same manner as I have described, and is kept in small cups, which are made for the purpose. The smoker lays his head on a pillow, has a lamp by his side, and with a kind of needle he lifts a small portion of the opium to the candle, and having ignited it, he puts it into the small aperture of the bowl of the pipe. The candle is applied to the bowl during the process of inhaling ; and the smoke is drawn into the lungs in the same manner as an Indian or Chinese swallows tobacco. A whiff or two is all that can be drawn from a single pipe, and, therefore, those who are accustomed to the use of the drug have frequently to renew the dose.

No one who has seen any thing of the habits of the Chinese will deny that the use of opium, particularly when taken to excess, has a most pernicious effect both upon the constitution and morals of its victims. From my own experience, however, I have no hesitation in saying that the number of persons who use it to excess has been very much exaggerated ; it is quite true that a very large quantity of the drug is yearly imported from India, but then we must take into consideration the vast extent of the Chinese empire, and its population of 300,000,000 of people. I have often been in company with opium-smokers when travelling in different parts of the country, and am consequently able to speak with some confidence with regard to their habits. I well remember the impressions I had on this subject before I left England, and my surprise when I was first in the company of an opium-smoker who was enjoying his favourite stimulant. When the man lay down upon the couch, and began to inhale the fumes of the opium, I observed him attentively, expecting in a minute or two to see him in his "third heaven of bliss ;" but no: after he had taken a few whiffs he quietly resigned the pipe to one of his friends, and walked away to his business. Several others of the party did exactly the same. Since then I have often seen the drug used, and I can assert, that in the great majority of cases it was not immoderately indulged in. At the same time I am well aware that, like the use of ardent spirits in our own country, it is frequently carried to a most lamentable excess. Lord Jocelyn, in his "Campaign in China," gives the following account of its effects, which he witnessed upon the Chinese at Singapore: "A few days of this fearful luxury, when taken to excess, will give a pallid and haggard look to the face, and a few months, or even weeks, will change the strong and healthy man into little better than an idiot or skeleton. The pain they suffer when deprived of the drug after long habit, no language can explain: and it is only when to a certain degree under its influence that their faculties are alive. In the houses devoted to their ruin, these infatuated people may be seen at nine o'clock in the evening in all the different stages; some entering half distracted to feed the craving appetite they had been obliged to subdue during the day: others laughing and talking wildly under the effects of a first pipe; whilst the couches around are filled with their different occupants, who lie languid with an idiot smile upon their countenance, too much under the influence of the drug to care for passing events, and fast merging to the wished-for consummation. The last scene in this tragic play is generally a room in the rear of the building, a species of dead-house, where lie stretched those who have passed into the state of bliss the opium-smoker madly seeks—an emblem of the long sleep to which he is blindly hurrying."—*Pharm. Jour., from Fortune's Wanderings in China.*

